# Therapeutic Evaluation of Antibody-Based Targeted Delivery of Interleukin 9 in Experimental Pulmonary Hypertension

**DOI:** 10.3390/ijms22073460

**Published:** 2021-03-27

**Authors:** Baptiste Gouyou, Katja Grün, Anne Kerschenmeyer, Alessandra Villa, Mattia Matasci, Andrea Schrepper, Alexander Pfeil, Laura Bäz, Christian Jung, P. Christian Schulze, Dario Neri, Marcus Franz

**Affiliations:** 1 Philochem AG, CH-8112 Otelfingen, Switzerland; Baptiste.Gouyou@philogen.com (B.G.); Anne.Kerschenmeyer@philogen.com (A.K.); Alessandra.Villa@philogen.com (A.V.); Mattia.Matasci@philogen.com (M.M.); neri@pharma.ethz.ch (D.N.); 2 Department of Internal Medicine I, Univerisity Hospital Jena, 07747 Jena, Germany; Katja.Gruen@med.uni-jena.de (K.G.); Laura.Baez@med.uni-jena.de (L.B.); christian.schulze@med.uni-jena.de (P.C.S.); 3 Department of Cardiothoracic Surgery, Univerisity Hospital Jena, 07747 Jena, Germany; Andrea.Schrepper@med.uni-jena.de; 4 Department of Internal Medicine III, Univerisity Hospital Jena, 07747 Jena, Germany; Alexander.Pfeil@med.uni-jena.de; 5 Division of Cardiology, Pulmonology, and Vascular Medicine, Medical Faculty, Heinrich-Heine University Düsseldorf, 40225 Düsseldorf, Germany; christian.jung@med.uni-uni-duesseldorf.de

**Keywords:** pulmonary hypertension, monocrotaline, mouse, fibronectin, immunocytokines, interleukin-9

## Abstract

Background and Aims: Pulmonary hypertension (PH) is a heterogeneous disorder associated with poor prognosis. For the majority of patients, only limited therapeutic options are available. Thus, there is great interest to develop novel treatment strategies focusing on pulmonary vascular and right ventricular remodeling. Interleukin 9 (IL9) is a pleiotropic cytokine with pro- and anti-inflammatory functions. The aim of this study was to evaluate the therapeutic activity of F8IL9F8 consisting of IL9 fused to the F8 antibody, specific to the alternatively-spliced EDA domain of fibronectin, which is abundantly expressed in pulmonary vasculature and right ventricular myocardium in PH. Methods: The efficacy of F8IL9F8 in attenuating PH progression in the monocrotaline mouse model was evaluated in comparison to an endothelin receptor antagonist (ERA) or an IL9 based immunocytokine with irrelevant antibody specificity (KSFIL9KSF). Treatment effects were assessed by right heart catheterization, echocardiography as well as histological and immunohistochemical tissue analyses. Results: Compared to controls, systolic right ventricular pressure (RVPsys) was significantly elevated and a variety of right ventricular echocardiographic parameters were significantly impaired in all MCT-induced PH groups except for the F8IL9F8 group. Both, F8IL9F8 and ERA treatments lead to a significant reduction in RVPsys and an improvement of echocardiographic parameters when compared to the MCT group not observable for the KSFIL9KSF group. Only F8IL9F8 significantly reduced lung tissue damage and displayed a significant decrease of leukocyte and macrophage accumulation in the lungs and right ventricles. Conclusions: Our study provides first pre-clinical evidence for the use of F8IL9F8 as a new therapeutic agent for PH in terms of a disease-modifying concept addressing cardiovascular remodeling.

## 1. Introduction

Pulmonary hypertension (PH) is a complex and heterogeneous disorder resulting from a variety of cardiovascular and respiratory diseases. PH is defined as an increase in mean pulmonary arterial pressure (PAPm) ≥ 20 mmHg at rest [1]. Current guidelines differentiate between five different groups depending on the underlying disease, which are of different prognostic relevance and define the appropriate treatment strategies: (1) pulmonary arterial hypertension (PAH), (2) PH due to left heart disease, (3) PH due to chronic lung disease and/or hypoxia, (4) chronic thromboembolic PH (CTEPH), and (5) PH due to multifactorial and unclear mechanisms [1,2,3]. Current treatment options for PH generally act by decreasing vascular tone and thereby reducing pulmonary artery pressure. Hitherto there is no therapeutic strategy capable to stop or even reverse the disease. Therefore, the presence of PH, especially when accompanied by right heart failure, is generally associated with poor prognosis and therapeutic options are limited for most patients. Indeed, whereas specific therapies are available for group 1 PH (PAH) (e.g., prostanoids, endothelin receptor antagonists, phosphodiesterase inhibitors), and group 4 PH (CTEPH) (soluble guanylate cyclase agonist), for the other groups, the standard of care is focused to symptom control or treatment of the underlying disease [3]. Consequently, mortality rates of PH patients are still high. Thus, there is a scientific and clinical need to identify novel therapeutic strategies applicable not only to group 1 PH (PAH) but also the much more frequently occurring groups 2 and 3. During PH development and progression, increased vasoconstriction and remodeling of the pulmonary vasculature occurs, resulting in PAP elevation, right ventricular pressure overload and the broad spectrum of clinical manifestations of the disease [2,4]. Pulmonary vascular remodeling, involving all layers of the vessel wall with an accentuation of the tunica media, is a key component of PH pathogenesis [2]. The cell adhesion modulating protein fibronectin represents an important component of the cardiovascular extracellular matrix and occurs as different molecular variants, which are generated by alternative splicing of the pre-mRNA leading to the inclusion or omission of so-called extra domains (ED) into the final multidomain protein. The latter show a disease-associated re-occurrence while being virtually absent in healthy adult tissues.

Recently, our group has identified extra domain A (EDA) (+)-fibronectin as a remodeling marker of PH in rats [5]. Interestingly, EDA overexpression has been reported in a variety of inflammatory conditions characterized by substantial tissue remodeling (e.g., endometriosis, arthritis, psoriasis, atherosclerosis, vasculopathy, and certain inflammatory bowel conditions) [6,7,8,9,10,11,12,13,14,15]. On the contrary, EDA is virtually undetectable in normal adult organs, with the exception of the female reproductive system [13]. Thus, EDA can be considered as an excellent molecular target for antibody-based pharmacodelivery of immunomodulatory payloads (e.g., cytokines) directly to the site of disease [7,13].

**Figure 1:**
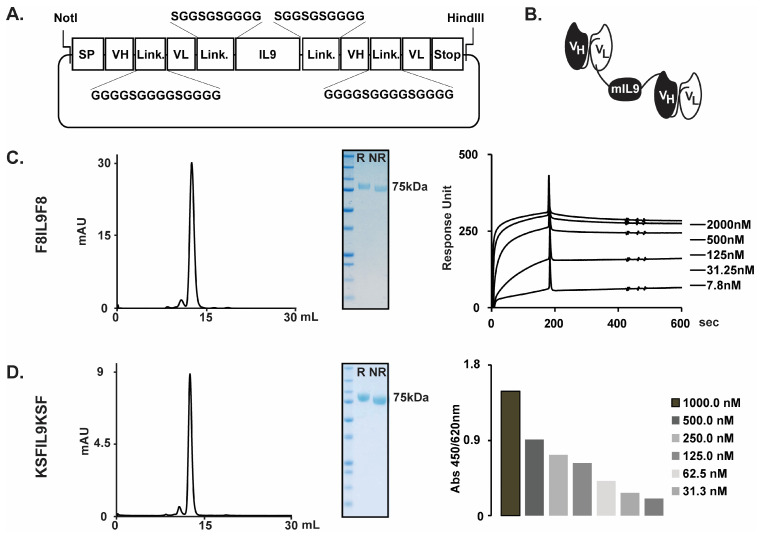
F8IL9F8 and KSFIL9KSF cloning expression and characterization. F8IL9F8 and KSFIL9KSF have been genetically engineered and produced in mammalian CHO cells. Cloning strategy of the IL9-based fusion proteins (**A**). Schematic representation of the domain structure of the IL9-based immunocytokines (**B**). Characterization of the F8IL9F8 (**C**) and KSFIL9KSF (**D**) purified proteins. From left to right: size exclusion chromatography profile, analytical SDS-PAGE analysis, SPR sensograms on EDA-coated CM5 chip for F8IL9F8, or ELISA on Lysozyme coated Maxisorp for KSFIL9KSF. R: reducing condition, NR: non-reducing condition SP: signal peptide, VH: variable heavy chain, VL: variable light chain, Link.: peptidic linker, STOP: codon stop.

Antibody-cytokine fusion proteins (i.e., immunocytokines) are increasingly being evaluated in preclinical and clinical settings for the treatment of cancer and chronic inflammation [7,8,9,10,13,14,16]. Our group has extensively investigated the use of the F8 fully human antibody, specific to EDA(+)-fibronectin, for the targeted delivery of a variety of cytokines (e.g., F8-IL10 and F8-IL4) at sites of chronic inflammation in mouse models [6,7,8,9,10,11] and in patients with rheumatoid arthritis [16]. Since murine and human EDA differ only in three amino acid substitutions, F8 is particularly suitable for in vivo pharmacodelivery applications because it recognizes the cognate antigen in mouse and man with identical affinity [17]. Interleukin-9 (IL9) is a pleiotropic cytokine mainly secreted by CD4+ T cells after stimulation by transforming growth factor-beta (TGFB) and IL4 [18]. IL9 signaling is mediated by a heterodimeric receptor consisting in the cytokine specific IL-9-receptor α-chain (IL-9Rα) dimerized with the common γ-chain receptor (γc) [19]. IL9 has been shown to activate T cells, eosinophils, type 2 innate lymphoid cells (ILC2), and mast cells [20,21]. Beside playing a regulatory role in autoimmunity and allergic reactions, IL9 has been suggested to be involved in anti-parasitic and anti-tumor responses, and in the formation of immune tolerance [22]. Whereas several biological functions have been attributed to IL-9, its involvement in PH has not been investigated so far, and results from preclinical models of lung fibrosis are controversial [23,24]. F8IL9F8 is a newly developed immunocytokine, consisting of one murine IL9 moiety flanked by two units of the F8 antibody in a single-chain Fv format (Figure 1). F8IL9F8 has been recently engineered to improve in vivo targeting efficacy. Consistent with the reported IL-9 anti-tumor activity [25], F8IL9F8 treatment resulted in mild tumor growth delay in murine models of melanoma and colon cancer [26].

In this study, we have evaluated the therapeutic efficacy of the recently developed immunocytokine, F8IL9F8, in the MCT-induced model of PH in mice. Disease severity has been evaluated by assessing haemodynamic and echocardiographic parameters as well as by ex vivo histological and immunohistochemical analysis of lung and heart sections to assess tissue damage and immune cell infiltrations. Surprisingly, different than the untargeted IL9 control (KSFIL9KSF), F8IL9F8 treatment exhibited a therapeutic advantage compared to the standard of care. While we are reporting a preliminary study on the therapeutic use of IL9 in PH, our findings suggest that F8IL9F8 is a potential candidate for the treatment of PH in terms of a disease-modifying concept addressing cardiovascular tissue remodeling.

## 2. Results

### 2.1. Production and Characterization of IL9 Based Immunocytokines

The F8IL9F8 and KSFIL9KSF fusion proteins consist of two scFv antibody moieties sequentially fused to the N- and C-termini of the murine interleukin 9, by the meaning of 10 amino acid long linkers. The fully human F8 antibody recognizes both the human and murine extra domain EDA of fibronectin with comparable affinity [17]. The KSF antibody (specific to the hen egg lysozyme) was used as a negative control antibody. The two fusion proteins were transiently produced in Chinese Hamster Ovary (CHO) cells at yields of about 20 mg/L. Figure 1A shows the cloning strategy chosen for the expression of the two immunocytokines, while Figure 1B illustrates the domain assembly of the corresponding polypeptides. The sequential arrangement of two scFv moieties to both termini of IL9 resulted in highly stable proteins with suitable properties for in vivo studies. F8IL9F8 and KSFIL9KSF were purified to homogeneity by protein A affinity chromatography and were well behaved in SDS-PAGE and size exclusion chromatography analysis (Figure 1B,C right panels). As expected, F8IL9F8 fully retained binding activity to the cognate EDA antigen as determined by Surface Plasmon Resonance (SPR), with an apparent KD of 2.9 10–9 nM. Similarly, KSFIL9KSF binding to the irrelevant antigen hen egg lysozyme was confirmed by ELISA (Figure 1B,C left panels).

### 2.2. Experimental Design of IL9 Treatment on MCT-Induced PH in Mice

**Figure 2:**
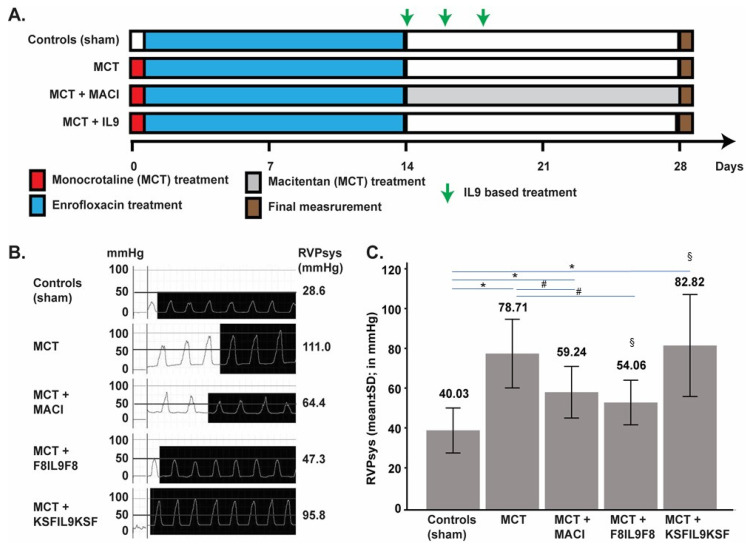
Treatment of MCT-induced PH mice with IL9 based fusion protein and MACI and effect on haemodynamic results after right heart catheterization. Experimental design of the MCT-induced PH mouse model and treatment schedule. Exception made for sham-induced controls (no PH), PH in mice was induced with a single injection of MCT, 60 mg/kg i.p. on day 0. From day 1 to 14, mice in all groups received Enrofloxacin 2.5% ad water, to prevent infection and inflammatory alterations of the lungs. Starting from day 14, mice received treatment with MACI (15 mg/kg daily) or IL9 based fusion proteins (200 µg every second day). On day 28, echography, RV-catheter, and organ harvesting were performed. Groups are composed by Sham (*n* = 6), MCT induced PH (*n* = 8), MCT induced PH + MACI (*n* = 7), MCT induced PH +F8IL9F8 (*n* = 6) and MCT induced PH + KSFIL9KSF (*n* = 6) (**A**). Representative right ventricular pressure (RVP) curves for one mouse per treatment group as recorded by invasive right heart catheterization. The second column shows the typical right ventricular morphology curve, and the third column provides the average cyclic maximum values (equates to systolic RVP = RVPsys) for each individual animal as calculated using LabChart software (in mmHg) (**B**). Chart representing the mean RVPsys (mean + standard deviation) of each treated group, performed by invasive right heart catheterization. Values presented on top of the graph are the mean value, calculated with LabChart software (in mmHg). Compared to the sham group, RVPsys was significantly elevated in all MCT-induced PH groups (*p* < 0.05; * in the bar graph), except for the group treated with F8IL9F8 (*p* = 0.068). F8IL9F8 and MACI treatments lead to a significant reduction in RVPsys (*p* < 0.05) when compared to the MCT group (# in the bar graphs). The group treated with F8IL9F8 displayed a significantly reduced RVPsys compared to the group treated with KSFIL9KSF (*p* = 0.047; § in the bar graphs) (**C**).

**Figure 3:**
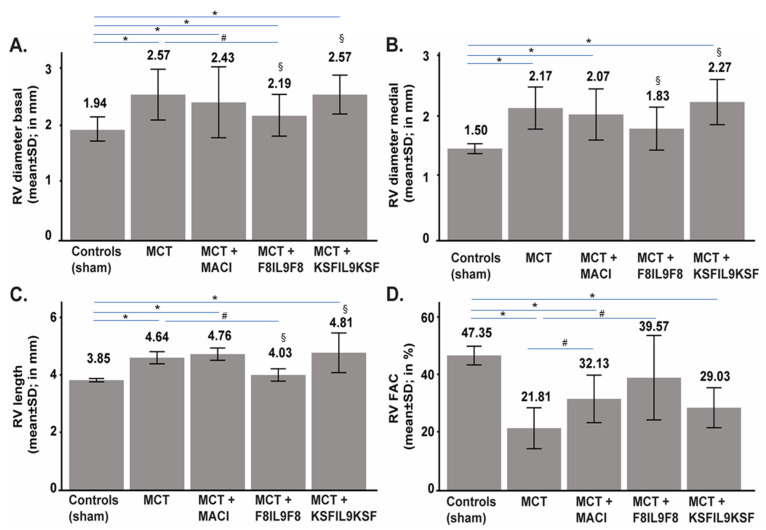
Echocardiographic assessment of treatment effect on MCT-induced PH mice. On day 28, echocardiography was performed in order to compare IL9-based fusion protein treatment to sham and MACI in MCT-induced PH mice. Echocardiographic measurement was performed using Vevo 770 Rodent-Ultrasound-System, Visual Sonic, Canada, 17 MHz probe RMV176. Values are given in comparison of the 5 experimental groups as mean ± standard in mm (RV basal and medial diameters as well as RV length) or in % (RV FAC), respectively. RV basal diameter was significantly increased in all MCT-induced PH groups (*p* < 0.05; * in the bar graph) compared to the sham group, only treatment with F8IL9F8 lead to a significant decrease of the RV basal diameter value compared to MCT-induced PH groups (*p* = 0.014; # in the bar graph). RV basal diameter in the F8IL9F8 group was significantly reduced compared to KSFIL9KSF treatment (*p* = 0.010; § in the bar graph) (**A**). The RV medial diameter in all the other MCT-induced PH groups was significantly increased compared to sham (*p* < 0.05; * in the bar graph) except of the F8IL9F8 group, in which the RV medial diameter was comparable to the one of the sham-treated group (*p* = 0.128). RV medial diameter in the F8IL9F8 group was significantly reduced compared to KSFIL9KSF treatment (*p* = 0.037; § in the bar graph) (**B**). RV length was significantly increased in all MCT-induced PH groups compared to sham (*p* < 0.05; * in the bar graph), except of the F8IL9F8 group (*p* = 0.100). In the F8IL9F8 group, RV length was significantly reduced compared to the untreated or KSFIL9KSF-treated groups (*p* = 0.006 and *p* = 0.016, respectively; # and § in the bar graph) (**C**). RV FAC values were significantly reduced in all MCT-induced PH groups compared to sham treated controls (*p* < 0.05; * in the bar graph) except the group treated with F8IL9F8 (*p* = 0.337). MACI and F8IL9F8 treatment showed a significant improvement in RV FAC compared to MCT-induced mice without treatment (*p* = 0.032 and *p* = 0.046, respectively; # in the bar graph) (**D**).Chart representing the basal (**A**) and medial (**B**) right ventricular diameter (RV diameter) values (mean ± standard in mm), right ventricular length (RV length) (**C**) values (mean ± standard in mm), and right ventricular fractional area change (RV FAC) (**D**) values (mean ± standard in %) in the 5 experimental groups.

Pulmonary hypertension could be successfully induced in C57BL/6 mice using the MCT method. 14 days after monocrotaline intraperitoneal (i.p.) injection, mice developed pulmonary hypertension with a suitable grade for therapeutic intervention. Mice were then treated either with (Macitentan) MACI (15 mg/kg daily), F8IL9F8, or its control KSFIL9KSF (both 200 µg/injection, three times every second day) (Figure 2A). Successful PH induction in the MCT induced mice was confirmed by the increase in right ventricular systolic pressure (RVPsys) and clear signs of right heart overload and dysfunction as assessed by echocardiography (Figure 2 and Figure 3). To determine PH severity in the different treatment groups, a variety of surrogate parameters of right ventricular (RV) morphology and function were assessed, including RV basal and medial diameters (in mm), RV length (in mm), and RV fractional area change (FAC, in %).

### 2.3. Effect of IL9 on Haemodynamic Parameter of MCT-Induced PH

The therapeutic efficacy of F8IL9F8 was tested in the MCT induced model of PH in mouse and compared both to the untargeted control KSFIL9KSF (an immunocytokine with irrelevant specificity in the mouse and therefore qualified as untargeted control), and MACI, a dual endothelin receptor antagonist proven to effectively reduce pulmonary artery pressures and therefore used as an approved standard therapy also in humans. Heart overload was assessed for each treatment group at 28 days after PH induction by recording RVPsys through right heart catheterization (Figure 2C). Compared to the sham group, RVPsys was significantly elevated in all MCT-induced PH groups (*p* < 0.05), except for the group treated with F8IL9F8 (*p* = 0.068). Both F8IL9F8 and MACI treatments lead to a significant reduction in RVPsys (*p* = 0.025 and 0.028, respectively) when compared to the MCT group. Additionally, the group treated with F8IL9F8 displayed a significantly reduced RVPsys compared to the group treated with KSFIL9KSF (*p* = 0.047). Therefore, the targeted delivery of IL9 by F8IL9F8 in the MCT murine model of PH is ameliorating right heart overload pressure in a manner similar to MACI treatment.

### 2.4. Effect of IL9 on Echocardiographic Parameters of MCT-Induced PH

Heart morphology and function were assessed at day 28 for each treatment group by echocardiography. RV basal and medial diameters, as well as the RV length, have been used as surrogate markers of RV-dilatation in response to pressure overload, while RV fractional area change was quantified and used as a surrogate marker of RV functions. While the RV basal diameter was significantly increased in all MCT-induced PH groups (*p* < 0.05) compared to the sham group, only treatment with F8IL9F8 lead to a significant decrease of the RV basal diameter value compared to MCT-induced PH groups (*p* = 0.014) (Figure 3A). F8IL9F8 treatment resulted in a RV medial diameter comparable to the one of the sham-treated group (*p* = 0.128), while in all the other MCT-induced PH groups, RV medial values were significantly increased compared to sham (*p* < 0.05). Importantly, both the RV basal and medial diameter values in the F8IL9F8 group were significantly reduced compared to KSFIL9KSF treatment (*p* = 0.010 for RV basal diameter and *p* = 0.037 for RV medial diameter) (Figure 3B). The RV length was significantly increased in all MCT-induced PH groups compared to sham (*p* < 0.05), except when mice were treated with F8IL9F8 (*p* = 0.100). Additionally, in the group treated with F8IL9F8, RV length diameters were significantly reduced compared to the untreated or KSFIL9KSF-treated groups (*p* = 0.006 and *p* = 0.016, respectively) (Figure 3C). RV systolic function was evaluated by calculating RV FAC values and by defining right ventricle dysfunction by RV FAC ≤ 35%. Compared to sham induced controls, the RV FAC values were significantly reduced in all MCT-induced PH groups (*p* < 0.05), except the group treated with F8IL9F8 (*p* = 0.337). Both MACI and F8IL9F8 treatments showed a significant improvement in the RV FAC values compared to MCT-induced mice without treatment (*p* = 0.032 and *p* = 0.046, respectively). Surprisingly, no significant differences in the RV FAC were observed between F8IL9F8 and KSFIL9KSF treatment (*p* = 0.068) (Figure 3D). In summary, also the echocardiographic parameters evaluated in this study demonstrate the therapeutic efficacy of F8IL9F8 for the treatment of PH in the MCT-induced murine model.

### 2.5. Effect of IL9 on Lung and Right Ventricular Cardiac Tissue Damage in MCT-Induced PH

#### 2.5.1. Lung Tissue Damage

**Figure 4:**
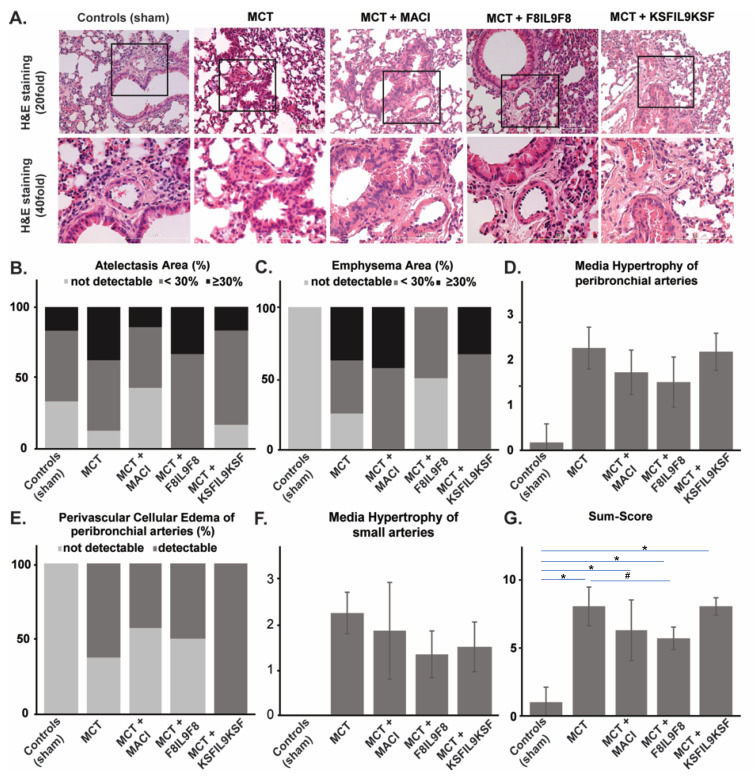
Assessment of histological damage in lung tissue: evaluation of different treatment effects on MCT-induced PH in mice. Lung tissue samples were subjected to detailed histological analysis to compare IL9-based fusion protein treatment to sham and MACI in MCT-induced PH mice. The assessment was performed by microscopic analysis using a defined sum-score system. Representative microscopic images of H&E, as well as Sirius red stains (**A**), illustrate the clear differences in the extent of tissue damage in comparison of the 5 experimental groups (**A**). Graphs show results of a semi-quantitative assessment of the following single parameters contributing to the sum-score for the different groups: atelectasis areas (%) (**B**), emphysema area (%) (**C**), media hypertrophy of peribronchial arteries (mean ± standard) (**D**), perivascular cellular edema of peribronchial arteries (%) (**E**), media hypertrophy of small arteries (mean ± standard) (**F**). The sum-score integrating evaluation results for all single parameters is given in (**G**) (mean ± standard). The sum-score was significantly increased in all MCT-induced PH groups compared to the sham group (*p* < 0.05; * in the bar graph). Only the F8IL9F8 led to a significant decrease of tissue damage when comparing to the MCT-induced PH group without treatment (*p* = 0.007; # in the bar graph) (**G**).

Tissue samples from the lungs and right ventricles of all 33 animals investigated in this study underwent detailed histological analysis, as described above. For lung tissue, a semi-quantitative sum-score system including all relevant histopathological parameters occurring in PH, was applied. The level of tissue damage in general (sum-score) was significantly increased when comparing the sham group with the MCT-induced PH groups (*p* < 0.05 for all groups). Most notably, when comparing the MCT-induced PH group without treatment with the treatment groups, only the administration of F8IL9F8 led to a significant decrease and thereby attenuation of tissue damage in the lungs (*p* = 0.007). Figure 4 shows representative histological phenomena (Figure 4A) and summarizes the results of semi-quantitative lung tissue analysis illustrating the sum-score (Figure 4G) as well as its single parameters (Figure 4B–F). When focusing on the latter, especially media hypertrophy of both peribronchial (Figure 4D) and small (Figure 4F) arteries, is significantly diminished exclusively in the F8IL9F8 group (*p* < 0.05). Comparison of MACI with F8IL9F8 treatment reveals a relevant decrease of emphysema area (*p* = 0.018). In the MACI treated group, perivascular cellular edema of peribronchial arteries was significantly decreased compared to the KSFIL9KSF group (*p* = 0.033). A comparison of the F8IL9F8 and the KSFIL9KSF group revealed a significant reduction in the emphysema area (*p* = 0.030), media hypertrophy of peribronchial arteries (*p* = 0.043), and the histological sum-score in general (*p* = 0.004) in the F8IL9F8 group.

#### 2.5.2. Right Ventricular Cardiac Tissue Damage

**Figure 5:**
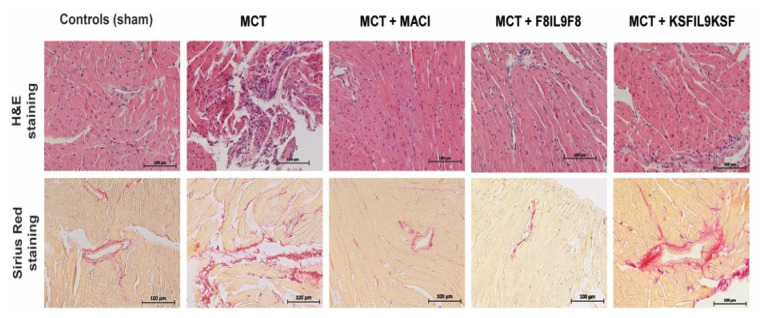
Histopathological changes in right ventricular cardiac tissue in comparison to the different experimental groups. Right ventricular cardiac tissue samples were subjected to histological analysis to compare IL9-based fusion protein treatment to sham and MACI in MCT-induced PH mice. Representative microscopic images of H&E, as well as Sirius red stains, illustrate the clear differences in the extent of tissue damage in comparison to the 5 experimental groups.

Histological analysis of right ventricular cardiac damage was performed with a focus on the assessment of inflammation and cardiac interstitial fibrosis by semi-quantitatively scoring as described above. In general, histological cardiac damage in the mouse model of MCT-induced PH is not as obvious as seen in the corresponding rat model (data not shown). When comparing the five different experimental groups in this study, there is a significant increase of both inflammation and fibrosis in the MCT-induced PH groups compared to the sham group (*p* < 0.05 for all groups except for inflammation in the group treated with MACI). When comparing the MCT-induced PH group with the under-treatment groups, there are no significant differences in the MACI as well as the F8IL9F8 group (*p* = n.s.) and a slight but significant augmentation of tissue damage in the KSFIL9KSF group (*p* < 0.05 for both inflammation and fibrosis). In the MACI group, compared to the KSFIL9KSF treated group, there was a significant reduction of inflammation (*p* = 0.045) and fibrosis (*p* = 0.043). Histopathological changes in right ventricular cardiac tissue in the comparison between the 5 experimental groups are illustrated in Figure 5.

### 2.6. Effect of IL9 on the Accumulation of Leukocytes and Macrophages in Lung and Right Ventricular Cardiac Tissue in MCT-Induced PH

**Figure 6:**
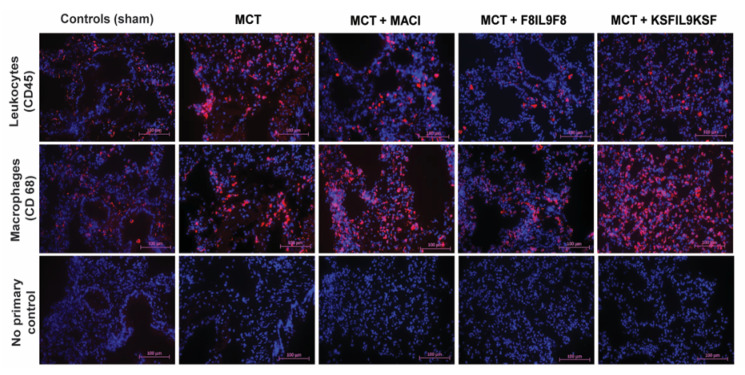
Assessment of leukocyte and macrophage accumulation in lung tissue: evaluation of different treatment effects on MCT-induced PH in mice. Lung tissue samples were subjected to immunofluorescence staining of CD45 (red fluorescence, DAPI staining in blue) as common leukocyte marker and CD68 (red fluorescence, DAPI staining in blue) as macrophage marker to quantitatively assess the extent of tissue accumulation to compare IL9-based fusion protein treatment to sham and MACI in MCT-induced PH in mice. The images in the lower row show the corresponding negative controls, in which the primary antibody was omitted. The magnification is 20 fold.

**Figure 7:**
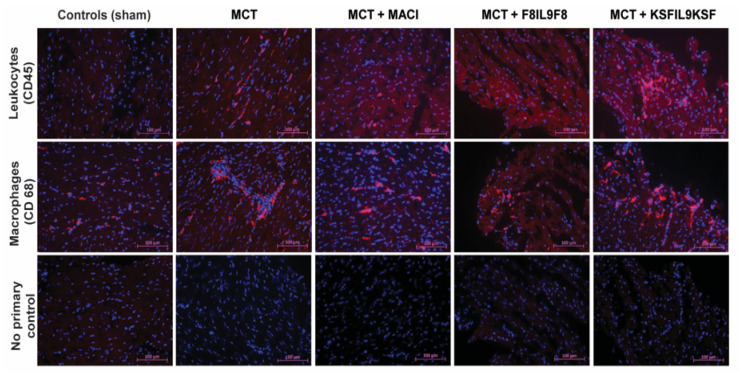
Assessment of leukocyte and macrophage accumulation in right ventricular cardiac tissue: evaluation of different treatment effects on MCT-induced PH in mice. Right ventricular cardiac tissue samples were subjected to immunofluorescence staining of CD45 (red fluorescence, DAPI staining in blue) as common leukocyte marker and CD68 (red fluorescence, DAPI staining in blue) as macrophage marker to quantitatively assess the extent of tissue accumulation to compare IL9-based fusion protein treatment to sham and MACI in MCT-induced PH in mice. The images in the lower row show the corresponding negative controls, in which the primary antibody was omitted. The magnification is 20 fold.

To further elucidate F8IL9F8 treatment effects on tissue inflammation, detection of CD45 as pan-leukocyte antigen and CD68 as a marker for macrophages was performed by immunofluorescence labeling. Representative samples of microscopic immunofluorescence detection results are given in Figure 6 for lung and in Figure 7 for right ventricular cardiac tissue. As compared to the sham-treated controls, there were significant increases of both leukocyte and macrophage accumulations in the lung and in the RV in all MCT-induced PH groups (i.e., untreated, F8IL9F8, KSFIL9KSF and MACI groups) (*p* < 0.05 for all). Compared to the MCT-induced PH untreated group, only the F8IL9F8 group displayed a significant decrease of macrophages and leukocytes accumulation both in lung and RV (*p* < 0.05 for all), whereas the MACI treatment group showed a significant decrease only in macrophage accumulation in the RV (*p* = 0.044), while no difference could be seen for the KSFIL9KSF treatment group (*p* = n.s. for all).

**Figure 8:**
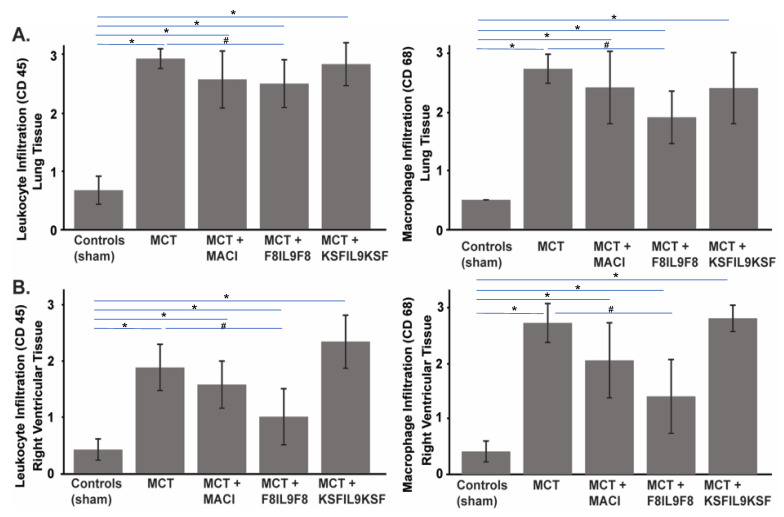
Results of semi-quantitative assessment of leukocyte and macrophage accumulation in lung and right ventricular cardiac damage to evaluate the different treatment effects on MCT-induced PH in mice. Graphs show results of the semi-quantitative assessment of leukocyte (CD45) and macrophage (CD68) accumulation in the lung (**A**) and right ventricular cardiac (**B**) tissue in comparison of the 5 experimental groups. Results are given as mean ± standard deviation. The assessment score ranged between 0 and 3 as described in the material and methods section. There were significant increases of both, leukocyte and macrophage accumulations in lung and RV in all MCT-induced PH groups compared to sham-treated controls (*p* < 0.05; * in the bar graphs). Only the F8IL9F8 group displayed a significant decrease of macrophages and leukocytes accumulation both, in lung and RV, compared to the untreated MCT-induced PH untreated group (*p* < 0.05; # in the bar graphs) (**A**,**B**).

Additionally, the KSFIL9KSF treatment group had a significant increase of leukocytes and macrophages accumulation in the RV, compared to the MACI treatment group (*p* < 0.05). This increase was even more significant when the KSFIL9KSF was compared to the F8IL9F8 treatment group (*p* < 0.05). However, no relevant differences could be noted for leukocyte and macrophages accumulation between the MACI and the F8IL9F8 treatment groups (*p* = n.s. for all). Figure 8 summarizes the results of the semi-quantitative assessment of leukocyte and macrophage accumulation in the lung (Figure 8A) and right ventricular cardiac (Figure 8B) tissue.

### 2.7. EDA(+)-Fibronectin Expression in Lung and Right Ventricular Cardiac Tissue in MCT-Induced PH Compared to Sham-Treated Controls in the Mouse Model

**Figure 9:**
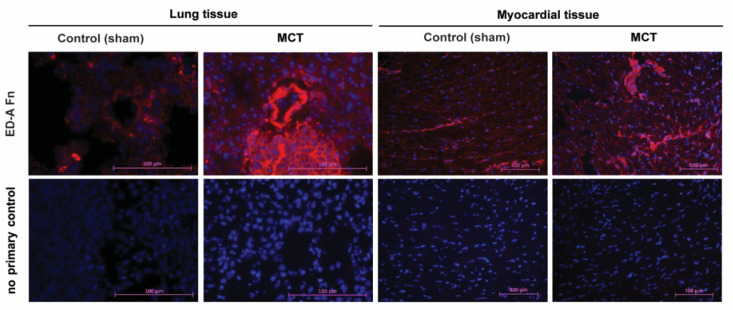
Expression of EDA(+) fibronectin as a specific target of the F8 antibody in MCT-induced PH compared to sham-treated mice. Lung and right ventricular cardiac tissue samples were subjected to immunofluorescence staining of EDA(+) fibronectin (red fluorescence, DAPI staining in blue) using the biotinylated antibody SIP-F8 in comparison of sham-treated and MCT-induced PH mice showing a strong up-regulation both with spatial accumulation to lung vessel structures and cardiac interstitial fibrosis. The images in the lower row show the corresponding negative controls, in which the primary antibody was omitted. The magnification is 40 fold for lung tissue and 20 fold for myocardial tissue.

The concept of F8 based targeted delivery of IL9 to pulmonary vascular as well as right ventricular cardiac tissue remodeling occurring in PH presumes the strong expression of EDA(+) fibronectin, which is the epitope specifically recognized by F8. As proven recently by our group in the rat model of MCT-induced PH [5], we could also demonstrate its strong re-occurrence in PH associated tissue damage in the corresponding mouse model used in this study. As compared to sham-treated controls, in MCT-induced PH, EDA(+) fibronectin is strongly expressed, and its tissue distribution shows, in particular, clear spatial associations to vessel structures in the lung and to cardiac interstitial fibrosis in the RV (Figure 9).

## 3. Discussion

In our study, we used an MCT-induced PH model in the mouse to evaluate the therapeutic efficacy of the novel immunocytokine F8IL9F8. The MCT model is an established animal model of PH, and the majority of drugs currently approved for the treatment of group 1 PH (PAH) were initially tested in this model [27,28,29].

Pulmonary hypertension is characterized by increased extracellular matrix turnover and pulmonary vascular remodeling [5,30]. Recently, we have identified EDA(+) fibronectin as a marker of pulmonary vascular and right ventricular cardiac remodeling in the MCT model of PH in rats [5]. Similarly, in our current study, we observed a strong EDA overexpression in lung and right ventricular tissues of MCT-induced mice. The specific deposition of EDA(+) fibronectin at PH lesions offers a perfect target for the antibody-based delivery of therapeutic payloads at the site of disease.

Our group has developed the F8 antibody, specific to the alternatively spliced EDA domain of fibronectin [17]. Over the past decades, we have produced a number of F8-based immunocytokines with different cytokine payloads (e.g., with IL2, IL4, IL6, IL10, IL12, TNF, interferon-alpha), many of which efficiently accumulate at the site of the disease, improving the therapeutic index of the corresponding cytokine payload [14,31,32,33,34,35,36]. In this study, we have used a recently described immunocytokine consisting of IL9 flanked by two moieties of the F8 antibody in single-chain Fv format [26]. As negative control, a non-targeted variant was generated using the irrelevant antibody.

There is increasing evidence demonstrating the role of cytokines and immune cells in the development and pathogenesis of PH, in particular, inflammation has been suggested as a predominant component of pulmonary vascular remodeling in various PH forms [30,37,38]. Multiple reports suggested a role of IL9 in lung inflammation and allergy [21,39,40,41]. For example, antibody-mediated neutralization of IL9 has been described to decrease lung inflammation and tissue damages caused by oxidative stress in a murine model of COPD [42] and to suppress lung injury and pulmonary fibrosis in mice that were intranasally exposed to silica [24]. Controversially, a reduction in alveolar fibrosis in IL9 overexpressing mice that were intratracheally treated with silica particles, has also been reported [23].

Therefore, we decided to test the therapeutic efficacy of the targeted delivery of IL9 in the MCT-induced PH mouse model. Disease severity was assessed by haemodynamic and echocardiographic parameters as well as by histological and immunohistochemical analysis of lung and right ventricular cardiac tissues.

Taking into account both, morphology and function of lung and heart, F8IL9F8 treatment demonstrated several obvious effects in PH mice compared to untreated controls or mice treated with the untargeted KSFIL9KSF. First, a significant reduction in RVPsys could be achieved associated with a significant improvement in right ventricle morphology and function. Furthermore, the histological analysis confirmed an attenuation of histological damage, in particular in lung tissue. In general, disease improvement mediated by F8IL9F8 was superior to the one observed in mice treated with the dual endothelin receptor antagonist (dual ERA), which belongs to the current standard of care medications for group 1 PH (PAH) [3].

In an attempt to understand the implication of immune response on the observed decrease in lung and heart tissue damage as well as hemodynamic and echocardiographic improvement by IL9 based therapy, immunofluorescence staining for CD45+ leukocytes and CD68+ macrophages was performed in lung and right ventricular tissue sections. While both, CD45+ leukocytes and CD68+ macrophages were increased in all MCT-induced PH groups compared to controls, they were both reduced in the groups treated with F8IL9F8 or the dual ERA MACI compared to the untreated or KSFIL9KSF treated groups. Moreover, compared to MACI, targeted IL9 delivery induced a stronger reduction in CD45+ leukocyte and CD68+ macrophages infiltration in the heart and in CD68+ macrophages in the lung. In line with the observed results, CD68+ macrophages have been reported to be abundantly present in the lungs of preclinical PH models and in PAH patients [38,43,44,45,46]. Moreover, it has been shown that inactivation or depletion of macrophages ameliorates PH conditions in different animal models [45,46]. Similarly, leukocytes are highly infiltrating the lungs of MCT-induced animals [47], and CD3+ and CD8+ cells are predominant in the lungs of PAH patients where they correlate with disease progression [48]. On the contrary, Treg activity may contribute to ameliorating PH disease. Athymic rats, which lack Tregs, are more susceptible to severe PAH than wild rats [49], while Treg immune reconstitution has been reported to be beneficial in a rat model in which PH was induced by the VEGFR2 antagonist SU5416 [50,51]. It has also been shown that an increased frequency of dysfunctional Treg cells is present in patients with PAH, which suggests that an altered immunosuppressive response may contribute to pulmonary vascular remodeling in PAH [52].

The observed reduction in infiltrating leukocytes and macrophages would suggest an anti-inflammatory role of IL9 in PH. However, a more detailed analysis of their subtypes, especially for M2 macrophages and Tregs, may provide further information on the mechanism by which F8IL9F8 diminishes PH severity. Indeed, IL-9 has been suggested to modulate the activity of macrophages by shifting their phenotype towards a more anti-inflammatory profile and by inducing their production of the anti-inflammatory TGFB [53,54]. Furthermore, IL-9 has been proposed to induce the resolution of inflammation in arthritis by promoting type 2 innate lymphoid cell (ILC2) -dependent Treg activation [55]. Since ILC2s are the dominant population of innate lymphoid cells in the lung [56], it would be interesting to investigate whether a similar mechanism may have contributed to the reduction of PH severity following F8IL9F8 treatment.

While additional studies are needed to clarify the mechanisms by which IL9 promotes amelioration of PH and to investigate the potential of F8IL9F8 to modify the pathological course of PH disease, the results presented in this study provide the first evidence that targeted delivery of IL9, mediated by the F8 antibody, to pulmonary hypertension lesions may represent a potential new treatment for PH.

## 4. Materials and Methods

### 4.1. Expression Plasmids

The plasmids pcDNA3.1-F8IL9F8 and pcDNA3.1-KSFIL9KSF were used for the production of the F8IL9F8 and KSFIL9KSF fusion proteins, respectively. The 2 vectors were generated by inserting into the HindIII-NotI sites of pcDNA3.1 (Invitrogen), a PCR assembled sequence consisting of the murine IL9 (AA 19–144) flanked on both N- and C- termini by either the F8 (raised against the EDA domain of fibronectin) [18] or the KSF (raised against the hen egg lysozyme) [57] antibodies in scFv format. In the assembled gene, the antibody moieties are fused to the IL9 cytokine by the meaning of 10 amino acids linkers. Figure 1A shows a schematic representation of the expression plasmids and the corresponding fusion protein.

### 4.2. Protein Expression and Purification

The IL9 based fusion proteins (F8IL9F8, KSFIL9KSF) were expressed by transient gene expression (TGE) in Chinese Hamster Ovary (CHO) cells [58]. For routine cultivation, cells were kept at 37 °C with a 5% CO_2_ atmosphere under shaking conditions. One day prior to transfection, CHO cells were seeded at 2 × 106 cells/mL in PowerCHO-2CD (Lonza) medium supplemented with 4 mM Ultraglutamine (Lonza). The next day, the cells were recovered by centrifugation and resuspended in ProCHO-4 Medium (Lonza) supplemented with 4 mM Ultraglutamine (Lonza) at a density of 4 × 106 cells/mL cells. Transfections were performed in glass bottles by adding 0.625 µg of plasmid DNA and 2.5 µg polyethyleneimine (1 mg/mL in water pH 7) (Polysciences) per million cells. The transfected cultures were maintained at 31 °C in a shaking incubator with 5% CO_2_ and with agitation at 120 rpm. The supernatant from 6 days old cultures was collected by centrifugation, and the fusion proteins were purified by a single step of Protein A affinity chromatography (Sino biological) and finally formulated in PBS via dialysis.

### 4.3. Protein Analysis

Dodecyl sulfate-polyacrylamide gel electrophoresis (SDS-PAGE) and size-exclusion chromatography Superdex 200 increase 10/300 GL column (Amersham Biosciences, Little Chalfont, UK) were used for characterization of protein size and purity. Analysis of the F8IL9F8 affinity to its cognate antigen was performed by Surface Plasmon Resonance (BIAcore X100, GE Healthcare, Arlington Heights, IL, USA). Samples were injected as serial-dilution (from 2000 nM to 7.8 nM) on a fibronectin 11A12 domain coated CM5 chip (GE Healthcare). ELISA was used to confirm the binding of KSFIL9KSF to hen egg Lysozyme. Briefly, hen egg lysozyme (Sigma, Darmstadt, Germany) was coated overnight at a concentration of 3 mg/mL on Maxisorp plates (ThermoFisher Scientific, Waltham, MA, USA). Captured KSFIL9KSF (triplicate of serial dilutions from 1000 nM to 31.3 nM) was detected with Protein A-HRP (GE Healthcare) using BM-Blue POD (Roche, Basel, Switzerland) as the substrate for horseradish peroxidase. Absorbance was measured at 450 nm vs 620 nm using a microplate reader (Ledetect 96, Dynamica Scientific, Portsmouth, UK). Results are expressed as an average of triplicate measurements.

### 4.4. Mouse Model of MCT-Induced PH and Treatment Schedule

All experiments were conducted according to the National Institute of Health Guidelines for the Care and Use of Laboratory Animals (8 th edition), to the European Community Council Directive for the Care and Use of Laboratory Animals of 22 September 2010 (2010/63/EU), the current version of the German Law on the Protection of Animals and the guidelines for animal care. The study protocol was approved by the appropriate State Office of Food Safety and Consumer Protection (TLLV, Bad Langensalza, Germany local registration number: UKJ17- 003). Animals were obtained from ZET facility (Zentale Experimentelle Tierhaltung) of the University Hospital Jena (UKJ, Jena, Germany). Prior to PH induction, mice were allowed to acclimatize for at least 7 days with ad libitum access to food and water as well as controlled light/dark cycles. PH was induced in C57BL/6 mice (bodyweight: 25–30 g), using the MCT method. As summarized in Figure 2 the following experimental groups were investigated: sham induced controls (*n* = 6), MCT induced PH (*n* = 8), MCT induced PH + MACI (*n* = 7), MCT induced PH + F8IL9F8 (*n* = 6), MCT induced PH + KSFIL9KSF (*n* = 6). The sham induced controls were injected with 30μL NaCl not containing MCT at day 1 (single dose intraperitoneally, i.p.). These mice did not develop PH and thus served as healthy controls. The other 4 experimental groups were injected with MCT to induce PH (single dose intraperitoneally, i.p. 60 mg/kg body weight volume 30μL). Animals in the “MCT + MACI” group received the drug from day 14 to day 28 (once daily per os 15 mg/kg body weight). Animals in the “MCT + F8IL9F8” group received F8IL9F8 3 times on day 14, 16, and 18 (intravenously, i.v. 200 μg/injection volume 155 μL). Accordingly, animals in the “MCT + KSFIL9KSF” group received KSFIL9KSF 3 times on day 14, 16 and 18 (intravenously, i.v. 200μg/injection volume 135 μL). To prevent infection and inflammatory alterations of the lungs, mice in all groups received Enrofloxacin (Orniflox) 2.5% ad water from day 1 to 14 after MCT injection. All animals were weighed and examined twice weekly for clinical monitoring of well-being. The clinical condition was assessed using an established score (clinical severity score = CSS) from 1 to 5 (1 = no signs of clinical alterations, 2 = low-grade impairment, 3 = mid-grade impairment, 4 = high grade impairment, 5 = exitus) obtained by evaluating spontaneous activity, reaction to exogenous stimuli and posture.

### 4.5. Echocardiographic Assessment

The echocardiographic assessment was performed on day 28 (Figure 3) using the Vevo 770 Rodent-Ultrasound-System, Visual Sonic, Canada, 17MHz probe RMV176. Before echocardiography, mice were anesthetized with isoflurane for a duration time of less than 10 min (isoflurane-CP, 2.5V%, FiO2 1.0, oxygen per inhalation-flow dosage). Body temperature and respiratory rate were continuously monitored. All surrogate parameters of right ventricular (RV) morphology and function were assessed, among others, RV basal and medial diameters (in mm), RV length (in mm), and RV fractional area change (FAC, in %).

### 4.6. Right Heart Catheterization

On day 28, after MCT injection, mice of all experimental groups were deeply anesthetized with a single dose of 100 mg/kg body weight Ketamine and 10 mg/kg body weight Xylazin in approximately 60 μL each administered i.p.. Right heart catheterization using a 1.4F micro conductance pressure-volume catheter (Model 10 SPR-839 Millar Instruments Inc PowerLab system, ADInstruments Ltd., Oxford, UK) was performed via the right vena jugularis interna to measure the systolic right ventricular pressure and thereby verify the success of the experimental setting. Mice were then sacrificed in deep anesthesia and analgesia to carry out cardiac blood collection after thoracotomy and to harvest the organs.

### 4.7. Histological Assessment of Lung and Right Ventricular Cardiac Tissue Damage

For the assessment of PH associated lung tissue damage, a defined sum-score system was used, which was recently established and validated in our group [5,59]. Microscopic evaluation was carried out using 4 μm thick H&E as well as Sirius Red stained lung tissue sections according to standardized protocols. The applied sum-score integrates the following histopathological features frequently occurring in PH: percentage of atelectasis area, percentage of emphysema area, degree of media hypertrophy of peribronchial arteries, presence of perivascular cellular edema of peribronchial arteries, and degree of media hypertrophy of small arteries. Finally, the scoring can range between 0 and 12, with the maximum value reflecting the highest level of tissue damage. Right ventricular cardiac tissue damage was evaluated by assessing the two most evident features, which are inflammation (immune cell infiltration) and cardiac interstitial fibrosis. Occurrence of these phenomena was semi-quantitatively assigned to the following levels: 0 = not detectable, 1 = weakly detectable, 2 = moderately detectable, 3 = strongly detectable. All histological analyses were performed independently and blinded by two experienced scientists.

### 4.8. Immunofluorescence Staining of CD45 (Pan-Leukocyte Antigen) and CD68

#### (Macrophage Marker) in Lung and Right Ventricular Cardiac Tissue

Immunofluorescence labeling of CD45 and CD68 was performed using 4 μm cryostat sections, which were fixed in ice-cold methanol (20 sec) and acetone (10 min). For the detection of CD45, the rat-anti-mouse monoclonal antibody 30-F11 (BD Biosciences, Heidelberg, Germany) was applied (dilution for lung tissue: 1:200 dilution for cardiac tissue: 1:150) and allowed to incubate for 60 min at room temperature. For the detection of CD68, the rat-anti-mouse monoclonal antibody FA-11 (Bio-Rad Laboratories, GmbH, München, Germany) was used (dilution for lung tissue: 1:200 dilution for cardiac tissue: 1:150) and allowed to incubate for 60 min at room temperature. As a next step, rinsing in TBS-T washing buffer was performed three times, and sections were then incubated with Cy3-conjugated AffiniPure Donkey Anti-Rat IgG (Jackson Immunoresearch Laboratories Inc., Pennsylvania, USA) for 45 min at room temperature (dilution 1:400). After rinsing in TBS-T buffer and distilled water, sections were mounted in Vectashield H1200 mounting medium containing DAPI (Linaris biologische Produkte GmbH, Wertheim-Bettingen, Deutschland, blue fluorescence) and stored at –20 °C. Antibody specificity control staining was performed in accordance but by omitting the primary antibodies. Immunofluorescence labeling was analyzed with the Axiophot microscope with the AxioVision Release 4.8 software (both Carl Zeiss, Germany). The magnification of microscopic images is 20-fold.

### 4.9. Immunofluorescence Staining of EDA(+) Fibronectin in Lung and Right Ventricular

#### Cardiac Tissue

For detection of EDA(+) fibronectin by immunofluorescence, again 4 μm cryostat sections were fixed in ice-cold acetone for 10 min. Blocking of endogenous biotin was performed with the DAKO Biotin Blocking System according to the manufacturer’s instructions (DAKO Deutschland,GmbH, Germany). The biotinylated SIP-F8 antibody was applied as a primary antibody in a dilution of 1:50 and allowed to incubate overnight at 4 °C. In the next step, Cy3 labeled Streptavidin (Southern Biotech, Birmingham, AL, USA) was added for 45 min in a dilution of 1:200 at room temperature. Antibody specificity control staining was performed in accordance but by omitting the primary antibodies (data not shown). Finally, sections were mounted using Vectashield H1200 containing DAPI-stain (Linaris biologische Produkte GmbH, Wertheim-Bettingen, Deutschland) and stored at −20 °C. Analysis of immunofluorescence labeling was performed by the Axiophot microscope with the AxioVision Release 4.8 software (both Carl Zeiss, Germany). The magnification of microscopic images is 40fold for lung and 20fold for cardiac tissue.

### 4.10. Statistics

Statistical analyses were performed using IBM SPSS statistical software, version 25.0 (IBM SPSS Statistics for Windows. Armonk, NY, USA). Data are expressed as mean ± standard deviation. Mann–Whitney-U test was used to test for significant differences between the different groups.

## Data Availability

The data presented in this study are available in this manuscript.
